# Detection of recombination events, haplotype reconstruction and imputation of sires using half-sib SNP genotypes

**DOI:** 10.1186/1297-9686-46-11

**Published:** 2014-02-04

**Authors:** Mohammad H Ferdosi, Brian P Kinghorn, Julius H J van der Werf, Cedric Gondro

**Affiliations:** 1 School of Environmental and Rural Science, University of New England, Armidale, Australia

## Abstract

**Background:**

Identifying recombination events and the chromosomal segments that constitute a gamete is useful for a number of applications in genomic analyses. In livestock, genotypic data are commonly available for half-sib families. We propose a straightforward but computationally efficient method to use single nucleotide polymorphism marker genotypes on half-sibs to reconstruct the recombination and segregation events that occurred during meiosis in a sire to form the haplotypes observed in its offspring. These meiosis events determine a block structure in paternal haplotypes of the progeny and this can be used to phase the genotypes of individuals in single half-sib families, to impute haplotypes of the sire if they are not genotyped or to impute the paternal strand of the offspring’s sequence based on sequence data of the sire.

**Methods:**

The hsphase algorithm exploits information from opposing homozygotes among half-sibs to identify recombination events, and the chromosomal regions from the paternal and maternal strands of the sire (blocks) that were inherited by its progeny. This information is then used to impute the sire’s genotype, which, in turn, is used to phase the half-sib family. Accuracy (defined as R^2^) and performance of this approach were evaluated by using simulated and real datasets. Phasing results for the half-sibs were benchmarked to other commonly used phasing programs – AlphaPhase, BEAGLE and PedPhase 3.

**Results:**

Using a simulated dataset with 20 markers per cM, and for a half-sib family size of 4 and 40, the accuracy of block detection, was 0.58 and 0.96, respectively. The accuracy of inferring sire genotypes was 0.75 and 1.00 and the accuracy of phasing was around 0.97, respectively. hsphase was more robust to genotyping errors than PedPhase 3, AlphaPhase and BEAGLE. Computationally, hsphase was much faster than AlphaPhase and BEAGLE.

**Conclusions:**

In half-sib families of size 8 and above, hsphase can accurately detect block structure of paternal haplotypes, impute genotypes of ungenotyped sires and reconstruct haplotypes in progeny. The method is much faster and more accurate than other widely used population-based phasing programs. A program implementing the method is freely available as an R package (hsphase).

## Background

Single nucleotide polymorphisms (SNPs) are the most common form of genetic variation. With the advent of new molecular technologies and the sequencing of many important species, a considerable amount of these markers can now be genotyped cheaply and precisely on a routine basis, not only for humans but also for livestock. In animal breeding, these markers are now used to estimate breeding values for commercially important traits.

Human genotypic data is usually sampled from a population of unrelated individuals or from trios, since the number of individuals in each family is small. However, in livestock populations, genotypes are often available from half-sib families. This data structure lends itself well to study the combinatorics that occurred during meiosis and allows identification of the recombination and segregation events that gave rise to the paternal haplotypes that are observed in the half-sib progeny. Once chromosomal segments (*blocks*) have been identified, they can be used to determine which progeny carry segments that are identical by descent (IBD), to phase the genotypes of the progeny or to impute sire or progeny genotypes, depending on whether the parent or the progeny has sparser marker data.

Generally, genotyping platforms do not provide phase information for the marker genotypes. Although advanced protocols have been developed that can generate phased genotype data directly, they are still too expensive for routine use in large-scale genotyping projects [1]. Instead, computational methods are used to infer the phase of marker genotypes. Phase information is important for association studies, where it can increase the power of the analyses. It can also provide valuable insights about the history of a population, be used to study signatures of selection, to estimate linkage disequilibrium, and to impute genotypes for genetic variants that have not been genotyped.

Various approaches have been developed to reconstruct haplotypes from genotype data and several phasing algorithms have been proposed [1-3]. Hickey et al. [4], in their AlphaPhase software, use the long-range phasing (LRP) method proposed by Kong et al. [5], combined with a haplotype library imputation method. If available, AlphaPhase uses pedigree information to partition surrogate parents into paternal and maternal surrogates. BEAGLE [6] and SHAPE-IT [7] use haplotype frequencies in addition to identity by descent (IBD) probabilities [1]. Windig et al. [8] use minimization of recombinations and all progeny information to phase the haplotypes. Li et al. [9] and Dajun et al. [10] phase haplotypes based on recombination minimization. Favier et al. [11] and Boettcher et al. [12] proposed a haplotype reconstruction method within half-sib families using a Monte Carlo approach and the likelihood of recombinations, respectively. The disjoint-set-structure (DSS) algorithm used in PedPhase 3 [13] is an effective algorithm that improves on the Integer Linear Programming approaches used in PedPhase 2 [9] and MERLIN [14] to reconstruct haplotype utilising pedigree information. Druet and Georges [15] proposed a heuristic method based on hidden Markov models that uses both population and family information to phase and cluster haplotypes. Another approach was suggested by Van Raden et al. [16], which partitions the chromosome into segments and phases marker loci for which an individual is heterozygous using information from homozygous loci. An extensive review of phasing strategies is given in Browning and Browning [1].

Different methods for haplotype reconstruction have different strengths and weaknesses. Currently, many widely adopted methods of phasing such as BEAGLE and SHAPE-IT make use of population-wide genotype data. For these methods, the accuracy of phasing is largely related to the number of samples, marker density, allele frequencies, population structure and quality of genotypes [1]. In livestock, family sizes are usually larger than in human populations and allele frequency distributions may be skewed by overrepresentation of some widely used individuals. Genotypic data on its own may not be sufficient to accurately reconstruct haplotypes and this is particularly true when the number of samples is limited. Pedigree information can then be used to increase phasing accuracy [2]. In addition, computing times and reliability of phasing results are important criteria for practical use [1] and will become even more so since the density of markers is rapidly increasing and routine use of full sequence data lies in the near future.

The aim of this study was to propose and evaluate a fast algorithm designed to identify recombination events in the sire of a half-sib family using SNP marker genotypes. This information can be used to identify recombination events. Here, we assume that genotypes are available on a group of paternal half-sibs, and the genotype of the sire is not required. The algorithm identifies which chromosomal segments each half-sib inherited from the paternal and maternal strands of the sire. This information is then used to impute the sire’s genotype, which can be useful in case the sire is not genotyped or if it is genotyped at a lower marker density, and to phase the genotypes of the half-sib progeny. Alternatively, if haplotyped sequence data is available for the sire, this method can be used to impute the paternal sequence in the haplotype that each offspring inherited, provided that the offspring is genotyped. Although restricted to half-sib data structures, this approach allows phasing of very small datasets (small single families) and can also be used to identify the location of recombination events or as a diagnostic tool to evaluate the accuracy of other haplotyping methods. A program (*hsphase*) that implements the method is freely available as an R package. The following sections describe the method, results with simulated and real datasets and compare its phasing accuracy to several other methods that are frequently used for phasing.

## Methods

Broadly, the method is based on exploiting the information content of opposing homozygous SNP marker genotypes and the linkage disequilibrium found within a half-sib family. Opposing homozygotes are markers for which one individual is homozygous for one allelic variant and another individual is homozygous for the other variant (here only bi-allelic variants are considered but the method can be extended to accommodate multi-allelic variants). First, all markers that have opposing homozygotes within a half-sib family are identified. At these markers, the paternal alleles are unambiguously identified and used to sort individuals into groups according to the paternal allele they received. The grouping of offspring at consecutive markers provides information about the most likely phase in the sire. We can then identify blocks of consecutive markers that were inherited together from the sire. This allows detection of likely recombination points, which can be visualized as a *block like* structure across the individuals’ genome where each *block* reflects the chromosome segment that the half-sib inherited from its sire. To illustrate this, consider that SNP genotypes are numerically coded as *0*, *1* and *2,* with *0* and *2* being homozygous and *1* heterozygous for a locus. For any given marker, if at least one half-sib has a *0* genotype and another one has a *2* genotype, the sire must be heterozygous at that locus and each half-sib progeny must have inherited one of the two allelic variants. To detect recombination between two neighbouring opposing homozygous sites in a pair of offspring, one offspring must have genotypes *2* and *2* at these loci and the other half-sib offspring must have a *0* and a *2* or, alternatively, one must be *0* and *0* and the other one has a *2* and *0*. Hence, a recombination is identified if the sum of the genotype codes at these loci across two half-sibs is equal to 2 or 6. The actual physical distance between these loci will depend on marker density and allele frequencies. The algorithm is detailed in the next section.

### Algorithm

#### Paternal strand detection based on opposing homozygous markers in half-sib families

**Figure 1:**
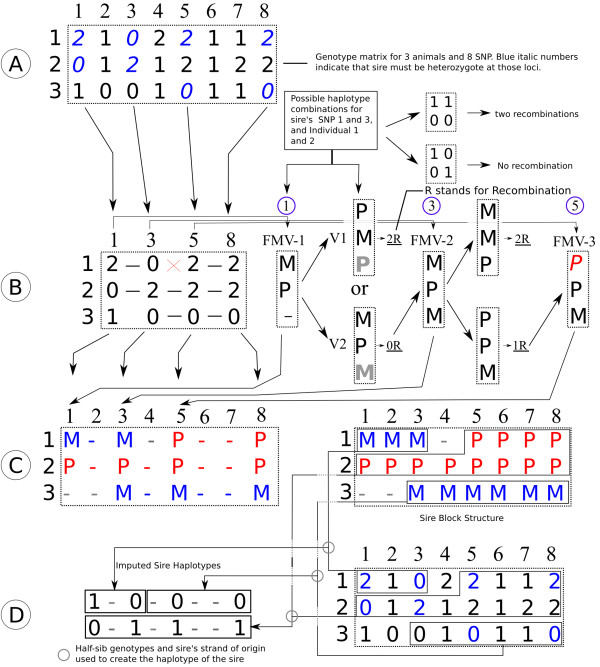
**Description of the hsphase algorithm for eight SNPs and three half-sibs. A** Genotype matrix (*0*, *2* are homozygotes, *1* are heterozygotes), blue genotypes highlight informative loci (opposing homozygotes – heterozygous in the sire, homozygous in the offspring). **B** Identification of the paternal origin of strands and recombination events (*x*) by using the *Forward Memory Vector*. (*P* and *M* are arbitrarily assigned paternal and maternal strands of the sire). **C** Final result of *blocking* after filling in the loci for which the sire strand could be determined. *P* and *M* refer to the two strands in the sire; - (dash) is used for unknown strand. **D** Imputation and phasing of the sire by combining the original offspring genotypes and the block structure.

The following algorithm was used to partition the genome of each half-sib progeny into blocks according to the haplotype it inherited from the sire. First, the markers for which the sire can be determined as heterozygous based on the offspring genotypes are identified (Figure 1A). Second, based on these markers, an empty matrix is created with dimensions equal to the number of half-sibs in the family by the number of markers. For the first locus we split the half-sibs into two *seed* groups based on the strand they inherited from the sire to provide an anchor from which to extend the blocks. For this purpose, we arbitrarily assign a “*P*” to progeny receiving the 0 allele and individuals that received the 1 allele are coded “*M*”. This loosely refers to paternal and maternal strands but note that the assignment is entirely arbitrary and *P/M* are simply used to distinguish between the two paternal strands; i.e. the strands of grand-parental origin inherited from the sire. Half-sib progeny that are heterozygous for this locus are recoded to unknown by entering the dash symbol (-).

A forward memory vector (FMV) is then created with the same number of rows as the number of half-sibs to temporarily store the grandparental origin codes (*P/M*) for this locus (Figure 1B). The FMV then steps to the next marker and, if there are no opposing homozygotes, the FMV codes of the previous locus are stored in the main *blocking* matrix for this marker column (Figure 1C). This is repeated until the FMV reaches the next opposing homozygous site (i.e. a marker for which both homozygous genotypes are present among the half-sib progeny). At this locus, the grand-parental origin of the paternal allele for individuals with opposing homozygous sites are determined and stored in vectors (e.g. Figure 1B, vectors **V**_**1**_ and **V**_**2**_). These vectors represent the two possible configurations of the grand-parental origin of the paternal allele. These vectors are compared to the FMV and the number of switches (recombinations) between each vector and the FMV is computed. The vector that needs fewer recombination events to explain the observed data is kept for the next step (e.g., in Figure 1B, vector **V**_**2**_ required less switches) and replaces the relevant values in the FMV, while the other genotypes (heterozygous sites) carry over as they were previously in the FMV. The values in the main matrix for the last locus are then updated accordingly (Figure 1C). This stepwise approach continues until the end of the chromosome is reached.

Recombination occurs when the minimum number of switches between FMV and the vectors (**V1** and **V2**) that are created from the next opposing homozygous locus is greater than zero. However, switches can also result from genotyping errors, which occur at a frequency of around 1% [17]. To avoid the identification of spurious recombination events due to genotyping errors, once a potential recombination has been identified for a particular individual, a temporary FMV is created to step through adjacent markers and validate the recombination event. This temporary FMV slides across a number of markers (e.g. 30) and as soon as three markers confirm the recombination, the process is interrupted and the recombination is deemed valid. If downstream markers do not validate the recombination (i.e. another recombination within a small genomic window is required to fit the observed marker genotypes), the recombination is considered to be due to a genotyping error and the original letters in the FMV for this marker are restored to nullify the recombination event. Each recombination event is evaluated in this manner.

At the end of the process, the *blocking* matrix stores the grandparental origin of the alleles at each locus, therefore showing the blocks that were inherited by each half-sib. The block information can be used to estimate linkage disequilibrium, the number and location of recombination events, to impute sire genotypes and to phase progeny genotypes.

#### Phasing and imputation of sire genotypes

**Figure 2:**
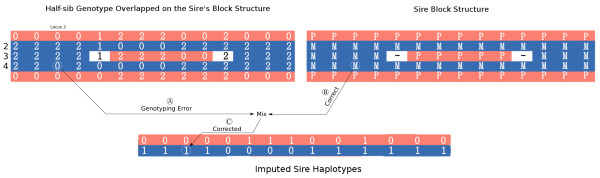
**Removal of genotyping errors. A** Genotyping errors in the half-sibs. A highlighted genotype is indicative of a genotyping error because only one marker supports its change to another block. **B** Fixing the genotyping errors in the half-sibs. The block structure is used to reject the recombination suggested by the genotype (it is not supported by downstream markers). **C** Fixing the genotyping errors in the imputed sire. Based on the blocking structure in the genotypes, individuals 2, 3, and 4 received the marker from the sire’s *M* strand (blue); the average number of markers (haplotype) in this location is 0.7, which is closer to 1 than 0 and this value is used as the sire’s imputed SNP genotype.

Sire haplotypes are inferred by simply averaging the sum of the genotypes at each marker of the half-sibs that inherited a particular strand (block) from the sire. These averages are recoded as *0* and *1* by rounding to the nearest integer and assigned to the sire’s haplotypes (Figure 1D). Albeit extremely simple, this approach is robust and computationally expedient, as detailed in the Results section. Genotyping errors are implicitly corrected for in the sire by selecting the most probable allele (Figure 2).

#### Phasing of genotypes of half-sib families

**Figure 3:**
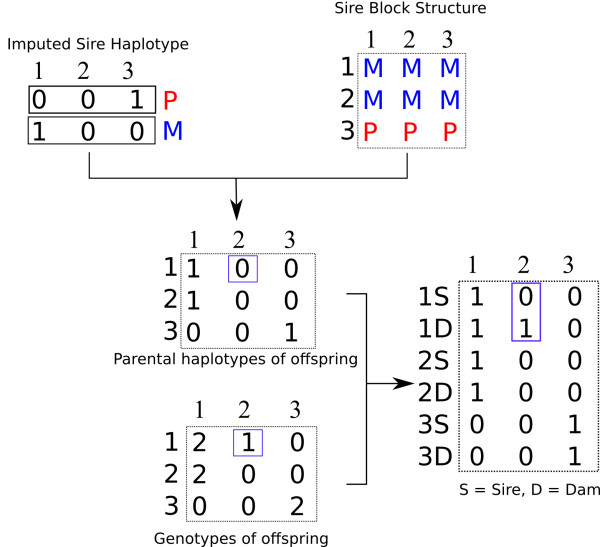
The sire’s imputed haplotypes and block structure information are used to phase the offspring.

Once the sire has been imputed and phased and the paternal strands have been partitioned across the half-sibs, the block matrix with parental origin codes (*P* and *M*) is then replaced by the paternal allele codes (0 or 1) pertaining to these origin codes. If the paternal origin code was uncertain, the origin was determined from the adjacent loci. The maternal haplotypes of the half-sibs are then obtained by simply subtracting the haplotype of a half-sib progeny from the sire from the individual’s genotype (Figure 3). Note that the parental strands for a given chromosome in the sire are arbitrarily assigned to be of “*P*” or “*M*” origin, so we cannot formally determine whether the paternal haplotype origin is grand-paternal or grand-maternal, but we can confidently assign a block structure to the paternal haplotypes that is consistent among the half-sibs within a family.

The algorithm is fully implemented as an open source R package. Implementation details are available from the source code.

### Estimation of phasing accuracy using strand of origin

#### Simulated datasets

Three simulated datasets (datasets A, B, and C) were generated to evaluate, benchmark and compare the algorithm with other commonly used phasing methods. Accuracy of phasing was evaluated as the R^2^ between *true* known haplotypes of the simulated sires and half-sibs and the haplotypes inferred by the phasing programs, where these haplotypes have binary values (0/1).

##### Dataset A

An R script was used to generate phased genotypes on a single chromosome for 500 000 SNP markers for a single sire and 400 half-sib offspring in order to evaluate runtimes of the algorithm on genotypes from high-density arrays. There were no genotyping errors. Allele frequencies were sampled from a uniform distribution U(0,1), and recombination rates were distributed as U(0,6).

##### Dataset B

This dataset was generated with the software QMsim that simulates data based on the population structure of commercial livestock animals [18]. We simulated a single chromosome of 500 cM, which is approximately 16% of the reported length of the sheep genome [19]. Three datasets were generated, each with sixteen generations and with, respectively, 1250, 5000 and 10 000 markers (for this setting ~8000 markers would be equivalent to the coverage of a 50K SNP chip). For each dataset, 20 males were mated to 400 females at each generation and genotypes were recorded for the last seven generations. Each genotype dataset used for analysis consisted of 120 half-sib families of 40 individuals. The genotypes of half-sibs from the first generation were not used because the genotypes of the sires from this generation were required to estimate the accuracy of sire imputation. The program QMsim generates phased genotypes, which allows results from the phasing algorithms to be compared to the *true* haplotypes for sires and offspring. To evaluate the robustness of the algorithms to genotyping errors, the 1% of the genotypes from these datasets was randomly selected using the *sample* function of R and randomly changed.

##### Dataset C

To compare the phasing component of our algorithm with other methods, QMsim was used to generate one population with the following structure: 10 generations, 1 chromosome of 500 cM and 10 000 markers, and 10 males and 200 females per generation. Only the genotypes from the last two generations were used and the genotypes of the sires from generation 8 were used to estimate the accuracy of imputation of the sires’ genotypes.

### Real dataset

Data generated by the SheepGenomics and the CRC for Sheep Industry Innovation on 4884 sheep genotyped with the 50K Illumina Ovine SNP chip and distributed across 110 half-sib groups were used. For expediency, after standard quality control filters, only data from chromosome 1 were used since it is the longest and has the largest number of markers (~5500). Data were from multiple breeds, the main breeds being Merino, White Suffolk, Border Leicester and Poll Dorset. Samples for genotyping were collected under approval number AEC12-049 of the University of New England Animal Ethics Committee.

### Performance comparison

BEAGLE 3.3.2 [6] and AlphaPhase 1.1 [4] are well known programs used for population-based phasing and were used to benchmark our approach. BEAGLE was run with default parameters and without pedigree information. AlphaPhase was also run with default parameters and with and without pedigree information. These methods require at least 1000 individuals to obtain adequate results; therefore all 1200 individuals from dataset C were used. PedPhase 3 [13] was used as a representative of pedigree-based phasing software that does not need large datasets. We ran PedPhase for all families, using single families of 20 half-sibs at a time (dataset C). Dataset A and C were used to compare computing times.

### Calculation of R^2^ and switch error rates

To compare phasing accuracies between programs, the R programming language was used to calculate the R^2^ between the phased and *true* haplotypes in the offspring for dataset C. We match both of the arbitrarily assigned paternal/maternal haplotypes with the correct simulated haplotypes, and selected the ones that gave the highest R^2^ per chromosome. Then, for all predicted haplotypes, the squared product moment correlation between them and the *true* haplotypes was calculated. Marker loci that could not be phased by any of the programs were omitted before calculating the R^2^ values.

Accuracy of phasing was also evaluated by calculating switch error rates (SWR). This was a function of the number of times an incorrect change of phase occurred in the inferred haplotype, divided by all possible switches. The mean of this number for each population was recorded and was calculated as:

$$\text{SWR}=\frac{\text{Number}\;\mathrm{of}\;\text{incorrect}\;\text{switches}}{\text{Number}\;\text{of}\;\text{heterozgous}\;\text{sites}-1}$$

### Implementation

For ease of use an R package was created which is freely available from the Comprehensive R Archive Network (CRAN) or from http://www-personal.une.edu.au/~cgondro2/hsphase.htm. R is the *de facto* standard language for statistical programming and is widely used in genetic analyses. To achieve high performance, the algorithm was written in C++. The R package includes several functions to run the analyses and to visualise and evaluate results.

## Results and discussion

### Simulated data

#### Computational cost

We initially tested our algorithm for speed using dataset A that consisted of a half-sib family with 500 000 markers and 400 individuals. For this example, the time needed for strand identification and phasing was ∼126 and ∼249 seconds respectively (running as a single thread on a 2.8 GHz computer; hsphase can make use of parallelization, which can significantly reduce this runtime). Runtimes scaled nearly linearly in the number of animals and the number of markers. The algorithm is very fast and simple to parallelize which makes it suitable for analyses of very dense marker panels and even for sequence data. Using dataset C (1200 individuals in 30 half-sib groups and 10 000 markers), we compared hsphase with other software programs. Population-based phasing took 42 minutes for AlphaPhase with pedigree, 157 minutes without pedigree and 46 minutes for BEAGLE. PedPhase 3 was used to phase half-sib groups one at a time with 20 half-sibs per family (PedPhase does not support families with more than 23 half-sibs), which required ~0.5 seconds per family (~30 seconds for the entire dataset). The program hsphase had a similar speed and took 19.5 seconds for the entire dataset. Based on these results, hsphase was orders of magnitude faster than AlphaPhase or BEAGLE and slightly faster than PedPhase 3. It should be noted that this comparison excludes the time needed for reading the genotypes into R, which adds an additional 3.2 seconds to hsphase, but this can vary significantly depending on the original format of the data.

#### Paternal strand detection

**Figure 4:**
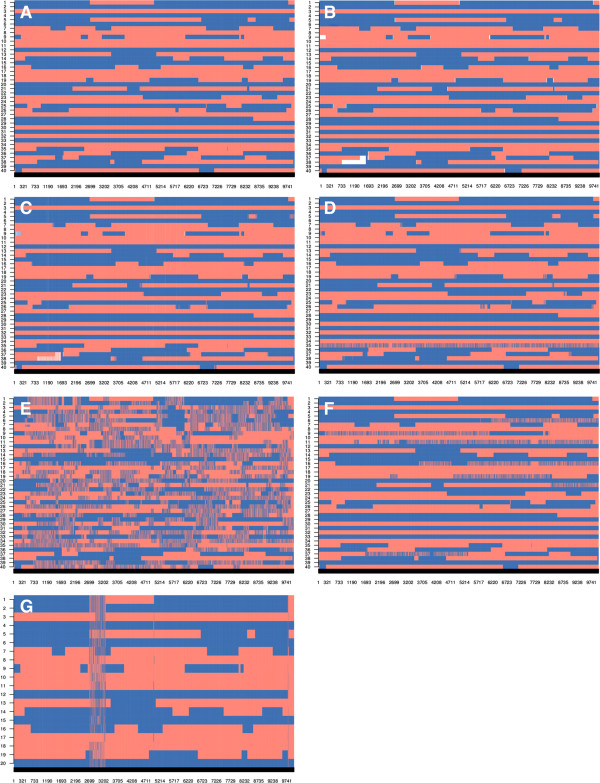
**Example of paternal strand blocking structure in a half-sib family with 40 individuals, using simulated data on 10 000 markers (dataset C). A**: true paternal strands of origin from the sire; **B**: strand assignment (blocks) with hsphase, empty spaces indicate unassigned regions; **C**: half-sib phasing with hsphase; **D**: half-sib phasing with AlphaPhase with use of pedigree; **E**: half-sib phasing with AlphaPhase without use of pedigree; **F**: half-sib phasing with BEAGLE; **G**: half-sib phasing with PedPhase; the red and blue colours indicate the paternal and maternal strands of the sire within each offspring, obtained by comparing the phased data with the sire’s true haplotypes; empty spaces indicate unknown strand or haplotype.

Figure 4A and B show the true and inferred strands of paternal origin (blocks) for 40 half-sibs and 10 000 markers (derived from dataset C). The figure shows a very high degree of agreement between true and predicted strands and few regions where the origin of the strands is unknown. The latter regions appear at recombination break points because non-informative markers between opposing homozygous markers cannot be resolved. The length of these unknown regions essentially depends on marker density and heterozygosity of the sire. The same results were achieved with the phased data (haplotype of sire and offspring - Figure 4C).

The R^2^ between true and detected strands was calculated to evaluate the accuracy of paternal strand detection. The value of R^2^ was very sensitive to errors related to phasing and identification of recombination events, but high values were achieved for most half-sib groups which indicate that the error rate was very low. Genotype errors, low-density markers and small half-sib groups had an adverse effect on the algorithm’s ability to correctly allocate grand-parental origin to the paternal chromosomal segments, and larger half-sib groups were required to give acceptable accuracy (R^2^ > 0.90) and get a clear blocking structure, i.e. a structure with a realistic number of recombinations and few segments where the correct paternal segments could not be identified. As expected, R^2^ increased and its standard deviation decreased as the number of half-sibs increased (Table 1).

**Table 1:** **R**^**2**^**+/- standard deviation across replicates between inferred and true results, and percentage of assigned results using simulated data (dataset B)**

	**Half-sib family size**					
	**4**	**6**	**8**	**10**	**20**	**40**
**1250 SNPs**						
**PS**	0*.*55 ± 0*.*37	0*.*65 ± 0*.*29	0*.*68 ± 0*.*27	0*.*77 ± 0*.*17	0*.*84 ± 0*.*07	0*.*84 ± 0*.*04
**PS%**	0*.*94 ± 0*.*13	0*.*96 ± 0*.*04	0*.*97 ± 0*.*02	0*.*97 ± 0*.*03	0*.*97 ± 0*.*03	0*.*98 ± 0*.*02
**PSe**	0*.*52 ± 0*.*36	0*.*64 ± 0*.*29	0*.*66 ± 0*.*27	0*.*75 ± 0*.*17	0*.*83 ± 0*.*07	0*.*83 ± 0*.*04
**PSe%**	0*.*96 ± 0*.*06	0*.*96 ± 0*.*04	0*.*97 ± 0*.*02	0*.*97 ± 0*.*03	0*.*98 ± 0*.*03	0*.*98 ± 0*.*02
**SI**	0*.*74 ± 0*.*25	0*.*81 ± 0*.*18	0*.*84 ± 0*.*17	0*.*90 ± 0*.*11	0*.*97 ± 0*.*03	0*.*99 ± 0*.*00
**SI%**	0*.*51 ± 0*.*09	0*.*71 ± 0*.*06	0*.*82 ± 0*.*05	0*.*88 ± 0*.*03	0*.*98 ± 0*.*02	1*.*00 ± 0*.*00
**SIe**	0*.*72 ± 0*.*23	0*.*80 ± 0*.*18	0*.*82 ± 0*.*18	0*.*89 ± 0*.*11	0*.*97 ± 0*.*03	0*.*99 ± 0*.*01
**SIe%**	0*.*53 ± 0*.*06	0*.*71 ± 0*.*06	0*.*82 ± 0*.*05	0*.*89 ± 0*.*03	0*.*98 ± 0*.*02	1*.*00 ± 0*.*00
**5000 SNPs**						
**PS**	0*.*55 ± 0*.*36	0*.*74 ± 0*.*32	0*.*86 ± 0*.*19	0*.*91 ± 0*.*07	0*.*93 ± 0*.*03	0*.*94 ± 0*.*02
**PS%**	0*.*95 ± 0*.*10	0*.*97 ± 0*.*03	0*.*98 ± 0*.*03	0*.*98 ± 0*.*02	0*.*98 ± 0*.*02	0*.*98 ± 0*.*02
**PSe**	0*.*56 ± 0*.*36	0*.*74 ± 0*.*33	0*.*87 ± 0*.*22	0*.*92 ± 0*.*12	0*.*95 ± 0*.*04	0*.*95 ± 0*.*02
**PSe%**	0*.*98 ± 0*.*03	0*.*98 ± 0*.*02	0*.*99 ± 0*.*02	0*.*99 ± 0*.*02	0*.*99 ± 0*.*02	0*.*99 ± 0*.*01
**SI**	0*.*74 ± 0*.*23	0*.*86 ± 0*.*20	0*.*93 ± 0*.*12	0*.*97 ± 0*.*04	0*.*99 ± 0*.*01	1*.*00 ± 0*.*00
**SI%**	0*.*50 ± 0*.*07	0*.*70 ± 0*.*07	0*.*81 ± 0*.*05	0*.*88 ± 0*.*04	0*.*98 ± 0*.*01	1*.*00 ± 0*.*00
**SIe**	0*.*71 ± 0*.*22	0*.*83 ± 0*.*20	0*.*91 ± 0*.*13	0*.*95 ± 0*.*05	0*.*99 ± 0*.*01	1*.*00 ± 0*.*00
**SIe%**	0*.*51 ± 0*.*04	0*.*70 ± 0*.*07	0*.*81 ± 0*.*05	0*.*88 ± 0*.*04	0*.*98 ± 0*.*01	1*.*00 ± 0*.*00
**10 000 SNPs**						
**PS**	0*.*58 ± 0*.*38	0*.*77 ± 0*.*32	0*.*90 ± 0*.*19	0*.*93 ± 0*.*10	0*.*96 ± 0*.*03	0*.*96 ± 0*.*02
**PS%**	0*.*95 ± 0*.*13	0*.*98 ± 0*.*02	0*.*99 ± 0*.*02	0*.*99 ± 0*.*02	0*.*99 ± 0*.*02	0*.*99 ± 0*.*01
**PSe**	0*.*48 ± 0*.*36	0*.*71 ± 0*.*33	0*.*84 ± 0*.*21	0*.*90 ± 0*.*09	0*.*92 ± 0*.*04	0*.*92 ± 0*.*04
**PSe%**	0*.*97 ± 0*.*04	0*.*98 ± 0*.*03	0*.*98 ± 0*.*03	0*.*98 ± 0*.*02	0*.*98 ± 0*.*02	0*.*98 ± 0*.*02
**SI**	0*.*75 ± 0*.*25	0*.*87 ± 0*.*20	0*.*95 ± 0*.*11	0*.*97 ± 0*.*06	1*.*00 ± 0*.*01	1*.*00 ± 0*.*00
**SI%**	0*.*50 ± 0*.*09	0*.*69 ± 0*.*06	0*.*80 ± 0*.*06	0*.*88 ± 0*.*06	0*.*99 ± 0*.*01	1*.*00 ± 0*.*00
**SIe**	0*.*75 ± 0*.*22	0*.*85 ± 0*.*20	0*.*93 ± 0*.*12	0*.*96 ± 0*.*06	0*.*99 ± 0*.*01	1*.*00 ± 0*.*00
**SIe%**	0*.*52 ± 0*.*06	0*.*69 ± 0*.*06	0*.*80 ± 0*.*05	0*.*88 ± 0*.*05	0*.*99 ± 0*.*01	1*.*00 ± 0*.*00

120 half-sib families with varying numbers of offspring per family (between 4 and 40) and different SNP marker densities were used; **PS**: paternal strand detection; **PS%**: percentage of markers assigned to paternal strands; **PSe**: paternal strand detection with 1% genotyping error; **PSe%**: percentage of markers assigned to paternal strands with 1% genotyping error; **SI**: sire genotype imputation, **SI%**: percentage of sire markers imputed; **SIe**: sire genotype imputation with 1% genotyping error, **SIe%**: percentage of sire markers imputed with 1% genotyping error.

Once the paternal chromosomal segments have been assigned, the blocking structure can be used for analysis of recombination events, e.g. detection of hot and cold spots, number of recombination events, extent of LD, etc. The resolution with which recombination can be resolved depends on the marker density and average distance between informative markers. It is possible that double recombination events are missed with this approach but this should be minimal with high-density SNP chips unless the sire’s heterozygosity is very low.

The image plots shown in Figure 2 can be generated using a function available in hsphase and are a useful visual diagnostic tool to evaluate the accuracy of strand detection and/or phasing. Recombination rates and the extent of LD are well established in most livestock species. Visual inspection of Figure 4E and 4F highlights that some individuals in the group appear to have a very large number of recombinations, which is not realistic. The main reason for a large number of recombinations observed in the sire is incorrect assignment of haplotypes in the offspring, i.e. haplotype blocks coming from the dam were assigned to the sire (e.g. animal 9 in Figure 4F); this generates a random scatter of switches between the two sire strands because that region did not originate from the sire. Inadequate phasing or a pedigree error shows a similar effect but, in those cases, random switches are seen across the entire chromosome instead of being compartmentalized into blocks. Finally, PedPhase 3 was unable to phase some loci that are in high LD (Figure 4G).

#### Sire genotype imputation

Dataset B was used to evaluate the accuracy of sire genotype imputation using the genotypes and paternal strand blocking structure that were identified in the half-sib groups. We tested the robustness of the algorithm by varying the number of individuals (between 4 and 40) per family. Since sire genotype imputation made use of information from multiple half-sib blocks, the accuracy was higher than the accuracy of strand allocation but was still directly related to correct block detection. As shown in Table 1, with families of size four, the accuracy of imputation was low at around 0.75 and only 50% of the markers were imputed correctly. For this case, increasing marker density had no effect on accuracy. With eight half-sibs per family, R^2^ and the number of markers correctly imputed ranged from 0.84 and 82% with 1250 markers to 0.95 and 80% with 10 000 markers. In this case, increasing marker density increased accuracy of sire imputation but not the percentage of markers that imputed correctly. With family sizes of 40 individuals, accuracies were essentially around 1.0 and 100% of the markers could be imputed. In general, marker density was more important when family size was at least six and with a family size greater than 20, the family information dominated and increasing marker density resulted in limited gains. Increasing the number of markers yielded quite significant improvements in accuracy when family size was around 10 offspring. Results in Table 1 indicate that a family size of eight was the threshold for accurate imputation of sire genotypes.

With genotyping errors of 1%, changes to accuracies and percentage of markers imputed were negligible (Table 1) [20]. Since the accuracy of imputation of sire genotypes was high, we consider that the imputed sire genotype can be applied as an additional quality control step to detect sire genotyping errors in real data when 20 or more half-sibs are available; sire imputation can also be used as a replacement for sire genotyping.

#### Half-sib phasing

The accuracy of half-sib phasing was high, even with very small family sizes, but with less than 10 half-sibs per family, the number of markers that could be phased decreased (Table 2). The latter was caused by a lack of opposing homozygous markers in the half-sibs to detect heterozygous sites in the sire. For family sizes between six and eight, the phasing accuracy dropped slightly because the number of individuals with ambiguous recombination sites increased (Table 2).

**Table 2:** **R**^**2**^**+/- standard deviation across replicates between inferred and true haplotypes**

	**Half-sib family size**					
	**4**	**6**	**8**	**10**	**20**	**40**
**1250 SNPs**						
**R**^**2**^	0.955 ± 0.034	0.923 ± 0.056	0.918 ± 0.048	0.913 ± 0.040	0.917 ± 0.036	0.934 ± 0.021
**SWR**	0.043 ± 0.035	0.055 ± 0.040	0.059 ± 0.037	0.057 ± 0.031	0.046 ± 0.020	0.034 ± 0.010
**HI%**	0.704 ± 0.056	0.815 ± 0.047	0.877 ± 0.044	0.903 ± 0.031	0.975 ± 0.008	0.985 ± 0.005
**R**^**2**^**e**	0.932 ± 0.035	0.896 ± 0.055	0.894 ± 0.052	0.888 ± 0.043	0.894 ± 0.033	0.907 ± 0.022
**SWRe**	0.075 ± 0.044	0.080 ± 0.037	0.079 ± 0.037	0.078 ± 0.030	0.064 ± 0.017	0.050 ± 0.010
**HIe%**	0.708 ± 0.054	0.821 ± 0.046	0.877 ± 0.041	0.907 ± 0.027	0.976 ± 0.007	0.987 ± 0.005
**5000 SNPs**						
**R**^**2**^	0.977 ± 0.026	0.969 ± 0.031	0.960 ± 0.029	0.961 ± 0.025	0.969 ± 0.014	0.975 ± 0.010
**SWR**	0.015 ± 0.018	0.018 ± 0.017	0.022 ± 0.009	0.023 ± 0.015	0.015 ± 0.009	0.008 ± 0.003
**HI%**	0.644 ± 0.159	0.800 ± 0.047	0.863 ± 0.037	0.900 ± 0.030	0.980 ± 0.009	0.990 ± 0.007
**R**^**2**^**e**	0.933 ± 0.057	0.934 ± 0.036	0.923 ± 0.029	0.925 ± 0.038	0.943 ± 0.015	0.947 ± 0.014
**SWRe**	0.044 ± 0.023	0.042 ± 0.015	0.045 ± 0.010	0.047 ± 0.018	0.032 ± 0.009	0.022 ± 0.003
**HIe%**	0.680 ± 0.044	0.806 ± 0.040	0.864 ± 0.032	0.905 ± 0.025	0.981 ± 0.010	0.991 ± 0.008
**10 000 SNPs**						
**R**^**2**^	0.977 ± 0.022	0.971 ± 0.025	0.958 ± 0.035	0.979 ± 0.013	0.977 ± 0.009	0.980 ± 0.009
**SWR**	0.012 ± 0.015	0.015 ± 0.007	0.018 ± 0.015	0.010 ± 0.006	0.007 ± 0.003	0.004 ± 0.001
**HI%**	0.680 ± 0.028	0.781 ± 0.035	0.867 ± 0.025	0.915 ± 0.032	0.983 ± 0.008	0.993 ± 0.004
**R**^**2**^**e**	0.954 ± 0.024	0.937 ± 0.027	0.926 ± 0.037	0.944 ± 0.030	0.948 ± 0.012	0.954 ± 0.011
**SWRe**	0.035 ± 0.015	0.038 ± 0.008	0.039 ± 0.017	0.029 ± 0.006	0.022 ± 0.004	0.017 ± 0.001
**HIe%**	0.682 ± 0.029	0.783 ± 0.036	0.871 ± 0.025	0.919 ± 0.029	0.986 ± 0.005	0.996 ± 0.001

Switch error rate (SWR) and percentage of assigned results (HI%) using simulated data (dataset B) on 120 half-sib families with varying numbers of offspring per family (between 4 and 40) and different SNP marker densities (1250, 5000 and 10 000); **e** suffix is for a 1% genotyping error.

The switch error rate (SWR) was more sensitive to the marker density. In the worst scenario (low-density markers, 1% genotyping errors and a family size of four), 92% of heterozygous sites were correctly phased with an SWR of 7.5%. With high-density markers (10 000 SNPs, 1% genotyping errors and a family size of four), the SWR was 3.5% and with a family size of 40 it was 1.7% but the R^2^ of haplotype inference for four and 40 half-sibs was nearly the same.

#### Comparison to BEAGLE, AlphaPhase and PedPhase

Dataset C was used to compare the hsphase algorithm with other methods in terms of phasing accuracy. The hsphase approach used information from only one half-sib family at a time, whereas AlphaPhase and BEAGLE used the information of all 1200 individuals across families because these methods rely on population parameters and therefore require larger datasets for satisfactory performance. PedPhase also used data from one family at a time but the program will not allow families with more than 23 individuals and also requires genotypes of the sire. Figure 4 illustrates how accurately the different programs allocated the sire’s strands to each half-sib. AlphaPhase with the use of pedigree information obtained very accurate results; with only a small overestimation of the number of recombination events as manifested by a limited number of short haplotypes (Figure 4D). The blocks were created from the haplotype of the sire and these haplotypes were generally correct, suggesting that the haplotype library method is efficient. AlphaPhase (pedigree free) considerably overestimated the number of recombination events for some individuals (Figure 4E). BEAGLE showed problems in particular regions, probably because part of the dam’s haplotype was assigned to the sire (Figure 4F). The accuracy of hsphase was also very high (Figure 4B), with better estimates of recombination events than other algorithms of haplotype reconstruction. The results show that hsphase gives accurate results with fewer incorrect indications of recombination, correct assignment of haplotypes between sire and dam, and correct assignment of blocking patterns in the paternal strands.

**Figure 5:**
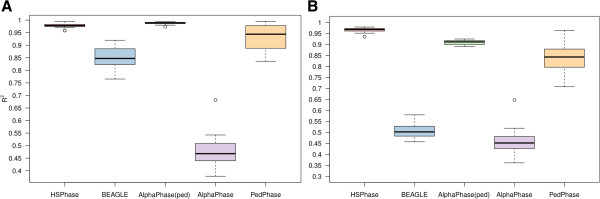
**Accuracy of haplotype reconstruction (hsphase, BEAGLE and AlphaPhase). A** Boxplot of *R*^2^ between inferred and true haplotypes for 20 half-sib families. Dataset C includes 10 000 SNP markers and 20 half-sib families with 40 individuals per family. **B** Boxplot of *R*^2^ between inferred and true haplotypes for the same data with 1% random genotyping error.

**Figure 6:**
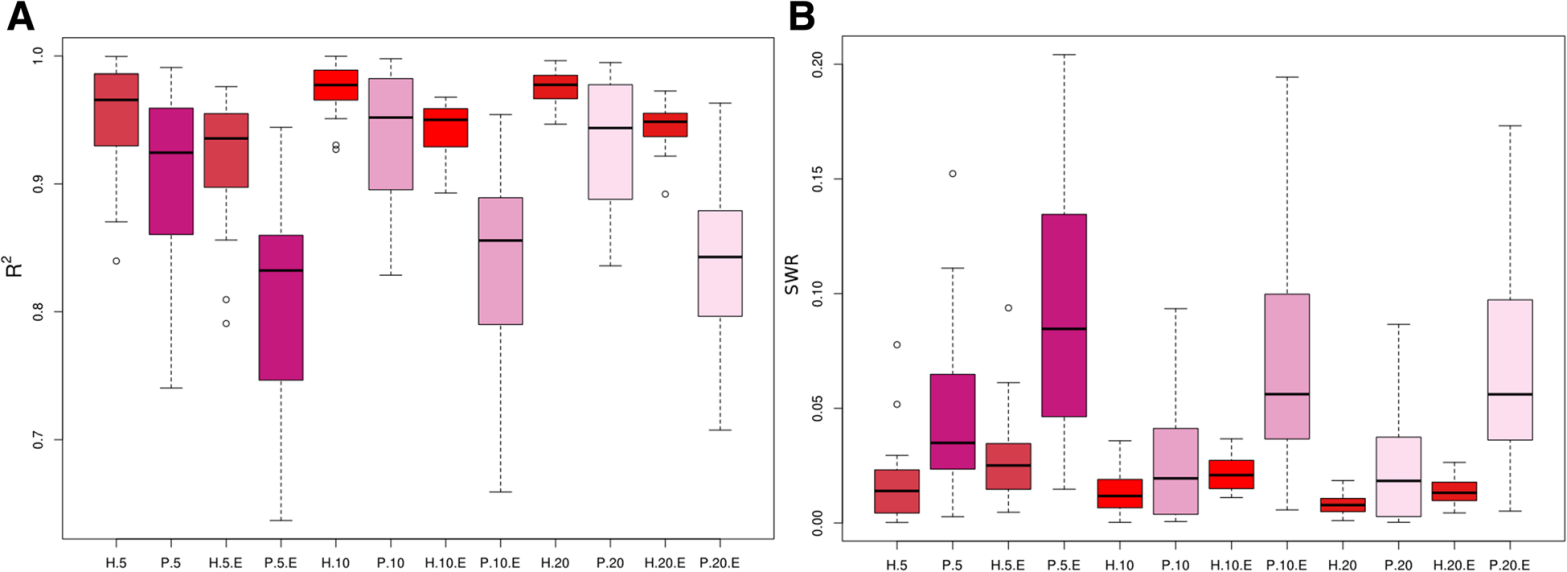
**Accuracy of haplotype reconstruction (hsphase and PedPhase). A** Boxplot of *R*^2^ between inferred and true haplotypes. **B** Boxplot of switch error rates. H = hsphase; P = PedPhase with 5, 10 and 20 half-sibs per family; E = with 1% random genotyping error.

The R^2^ obtained by randomly phasing dataset C was ~0.28 (based on the mean of 10 000 repeats), which sets the baseline for comparison of the different methods. The R^2^ of AlphaPhase with use of pedigree and hsphase were nearly the same. The R^2^ of AlphaPhase without use of pedigree was less than 0.5 (Figure 5A). The reason for this low R^2^ is that one switch (incorrect recombination) in the middle of a strand can affect the phasing of the rest of the strand. The same effect was observed with BEAGLE (Figure 5A) but to a lesser extent. Genotyping errors of 1% only had a moderate effect on hsphase (Figure 5B) but a larger effect on the other methods. However, if the population is large and includes a considerable number of small half-sib groups with reliable pedigree data, AlphaPhase is recommended over hsphase since hsphase cannot accurately phase small half-sib groups. However, when family sizes are larger than 10 half-sibs, hsphase is preferred since it is much faster and more accurate than the other methods, particularly when the total number of genotypes is small. PedPhase had a higher R^2^ than AlphaPhase and BEAGLE but this method gave more variable results, probably driven by the fact that all markers are phased by this method (Figures 5A and 6A). Genotyping errors reduced the accuracy of PedPhase and further increased variability. PedPhase requires that the genotypes of the sire are included in the analysis in order to use a pedigree approach.

The switch error rate (SWR) was low for all methods, ranging from 0.01 to 0.05%, except for PedPhase, which had an SWR of ~0.5%. This suggests that these population-based methods as well as hsphase are all good at phasing over smaller chromosome segments and this can be useful for the purpose of QTL (Quantitative Trait Loci) mapping or in case–control studies.

As mentioned above, it is also possible to use the phased half-sib data to create a block structure, which is useful to evaluate the algorithms based on their power to infer these blocks, both visually and by counting the number and distance between recombination events in each individual. Based on length of the chromosome, a certain number of recombination events are expected. An unrealistic number of recombinations in the blocking structure provide evidence of either a pedigree error or algorithmic problems. Overall, we observed an improvement in accuracy with hsphase compared to other methods, particularly with 1% genotyping errors (Figure 5B). With the simulated datasets, we could use the image plots in Figure 2 to compare the inferred blocks of phased results with the true sire haplotypes. However, even with real data, for which the sire haplotypes are unknown, the inferred blocking patterns can be used to evaluate phasing algorithms. It should be noted here that both AlphaPhase and BEAGLE make extensive use of a wide range of parameters such as number of surrogates, percentage surrogate disagreement, and number of iterations and so on. The accuracy obtained with these methods could probably be increased if the choice of parameters was optimized for these datasets. The image plot and the number of recombinations per individual can be useful to identify the optimal phasing parameters.

### Real data

#### Paternal strand detection

**Figure 7:**
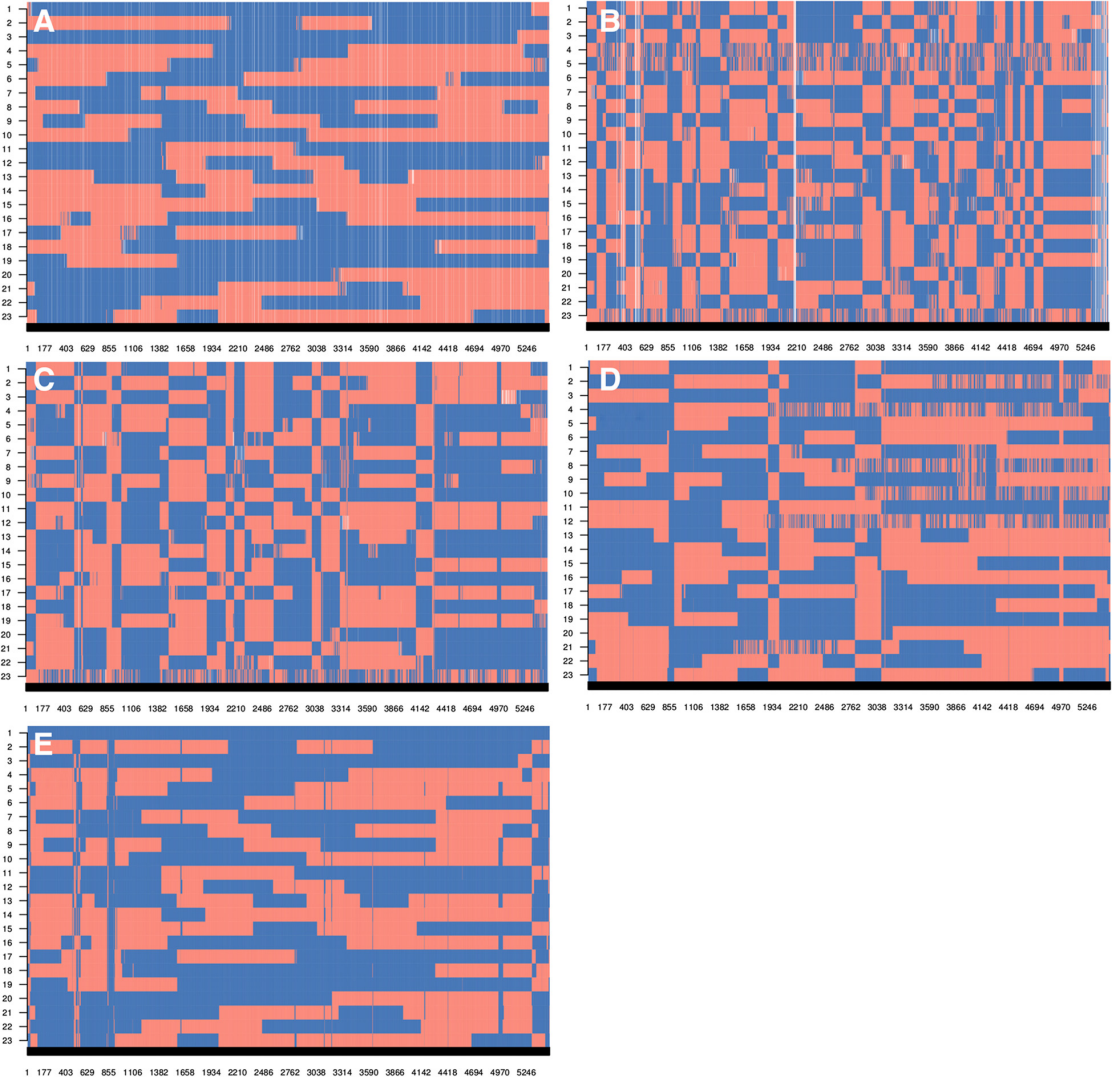
**Example of paternal strand blocking structure built from a phased half-sib family with 23 individuals using real data genotyped on the 50K Ovine Illumina array. A**: hsphase; **B**: AlphaPhase with pedigree; **C**: pedigree free AlphaPhase; **D**: BEAGLE; **E**: PedPhase; red and blue colours indicate the sire’s strands inherited by each offspring, obtained by comparing the phased data with the sire’s phased haplotypes, as inferred by the respective methods (the sire’s genotypes were part of the dataset); empty spaces indicate unknown strand or haplotype.

Figure 7 shows the sire’s blocking structure that was inferred by the different methods for a phased half-sib family with 23 individuals from the real dataset. Results were consistent with the simulated data (Figure 4) and both BEAGLE and AlphaPhase without the use of pedigree showing too many recombination events. The blocking structure inferred by hsphase (Figure 7A) was biologically realistic since it had a plausible number of recombination events and the recombinations occurred not too close to each other. The recombination events can be identified based on the block structure and this can be visualised for the half-sib group as done in Figure 7. AlphaPhase, BEAGLE and PedPhase showed excessive numbers of recombination events in some individuals (Figures 7B,C,D,E). Therefore, hsphase seems to be the only one that is able to give a reliable indication of the recombination events.

#### Imputation accuracy of sire genotypes

**Figure 8:**
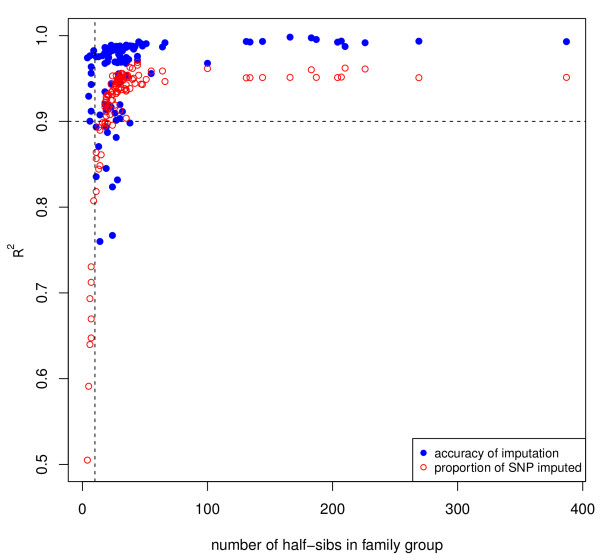
**Accuracy of sire imputation utilising real data.***R*^2^ values (in blue) between imputed and observed sire genotypes (for sheep chromosome 1), with different numbers of half-sibs per family (110 groups, mean = 0.95, median = 0.98) and percentage of imputed SNPs (in red) in the sires. The vertical line is for families of size 10.

The accuracy of imputation of sire genotypes with hsphase was evaluated using all 110 half-sib families and compared to the true genotypes of the sire. The R^2^ between observed and imputed sire genotypes had a mean of 0.95 and a median of 0.98 (Figure 8). For families with less than 10 half-sibs, the number of marker genotypes that could be imputed decreased dramatically (Figure 8). The maximum accuracy obtained for sire imputation in real data was around 97%. Genotyping errors prevent a 100% accuracy of imputing the sire genotype. By comparison, the accuracy of imputation of sire genotypes with hsphase was around 99% in simulated data when the half-sibs family size was larger than 20 and with 1% genotyping error.

## Conclusions

In this work, we proposed a simple method that relies on linkage disequilibrium within families and uses loci with opposing homozygotes within a half-sib family to identify paternal chromosomal segments and recombination events. The resulting paternal *blocking* structure can be used for phasing and imputation of sire genotypes. An R package *hsphase* that implements the method has been made available. The computational speed of the algorithm allows it to be used on large datasets. The accuracy of blocking and sire imputation of the algorithm was high when eight or more half-sibs were available. Imputation of sire genotypes was accurate and might eliminate the need to genotype the sire. For phasing, even a single family of four individuals had an R^2^ above 0.95 but the percentage of markers phased was ~70%. In addition, it was shown that the blocking structure derived from the paternal strand of origin is a valuable diagnostic tool to quantify and detect phasing and pedigree errors irrespective of the phasing method employed. Lastly, we suggest that if sequence or high-density marker data is available for the sire, the blocking structures lend themselves to imputation of the paternal strand in half-sib family groups.

## Competing interests

The authors declare that they have no competing interests.

## Authors’ contributions

MF and CG designed the algorithm and experiments to test it. JHJW and BPK advised on the design of the experiments to test the algorithm. All authors read and approved the final manuscript.
